# Short telomeres and severe COVID-19: The connection conundrum

**DOI:** 10.1016/j.ebiom.2021.103513

**Published:** 2021-07-29

**Authors:** Abraham Aviv

**Affiliations:** The Center of Human Development and Aging, New Jersey Medical School, Rutgers University, Newark, New Jersey, 07103 USA

The exponential rise of COVID-19 mortality among older adults has been rightly attributed to a declining immunity with age. Still, for unknown reasons, older adults are far more likely to die from COVID-19 than from the seasonal influenza, and this puzzle demands specific answers. One of them emerges from the telomere study by Wang et al [1]. Other small studies [2,3], each less than 100 participants, have also observed association of short hematopoietic cell telomeres with severe COVID-19. Wang's study, in contrast, consists of 6,775 adults with COVID-19. The study's importance, however, rests not in its size but in the fact that hematopoietic cell telomere length (TL) measurements had been performed years before the SARS-CoV-2 pandemic. This critical fact means that short hematopoietic cell TL, as expressed in leukocyte TL (LTL), preceded the onset of severe COVID-19– a conclusion that excludes reverse causality, namely, that SARS-CoV-2 infection shortens LTL.

The TL variation within the individual's somatic cells is much smaller than the inter-individual TL variation. Accordingly, LTL has been used as a proxy for TL in leukocyte lineages and other somatic cells [4]. That is relevant for another puzzling feature of COVID-19, i.e., the infection is often associated with lymphopenia [5]. Transient lymphopenia is commonly observed in individuals with acute viral infections, but COVID-19 lymphopenia is atypical in its severity and long duration. This lymphopenia principally results from plummeting counts of T-cells, i.e., it is T-cell lymphopenia [5,6].

The T-cell blood pool reflects a homeostatic balance between T cell depletion due to a host of factors and T-cell repletion, which is principally accomplished in adults through T-cell clonal expansion. Telomerase, the reverse transcriptase that elongates telomeres, is activated during T-cell clonal expansion and differentiation, but the activity of the enzyme is insufficient to counter telomere shortening with T-cell replication. T-cell replication is thus TL-dependent [7]. As hematopoietic cell TL shorten with age, T-cells from older adults have diminished clonal expansion capacity compared to young adults. For most healthy adults, such age-dependent T-cell TL shortening might not be a major problem in absence of an acute infection, because the long half-lives of ~ 5 years of naïve cells and ~ 5 months of memory T cells in the circulation impose minimal demand on the clonal expansion capacity of T-cells [8]. However, a massive and rapid T-cell clonal expansion might be needed after contracting SARS-CoV-2 infection.

The primary cause of COVID-19 T-cell lymphopenia is still poorly understood. We do know, though, that a proper T-cell clonal expansion in response to SARS-CoV-2 infection is crucial– so crucial that without it surviving the infection is at stake. This clonal expansion is necessary not only for reversing the downwards T-cell count trajectory towards a normal T-cell count; it is also essential for forming SARS-CoV-2 antigen-specific effector/memory T cells that clear the virus. Short naïve T-cell telomeres might thus limit adaptive immunity against SARS-CoV-2 even without a clinical manifestation of T-cell lymphopenia.

Sette and Crotty have recently suggested that a poor T-cell response in patients with COVID-19 leaves the innate immune response relatively unchecked [6], which might result in hyper-inflammation in the form of a cytokine storm and severe lung injury. Relatedly, short hematopoietic cell TL is associated with severe pulmonary fibrosis post-COVID-19, pointing to a potential link between short T-cell TL and increased pulmonary injury during the infection [3]. Jointly, these findings are consistent with a telomere model (Fig. 1) that explains the mortal threat of SARS-CoV-2 infection to individuals with short hematopoietic cell telomeres, particularly older adults whose telomeres are much shorter than those of younger adults and children.

**Fig. 1:**
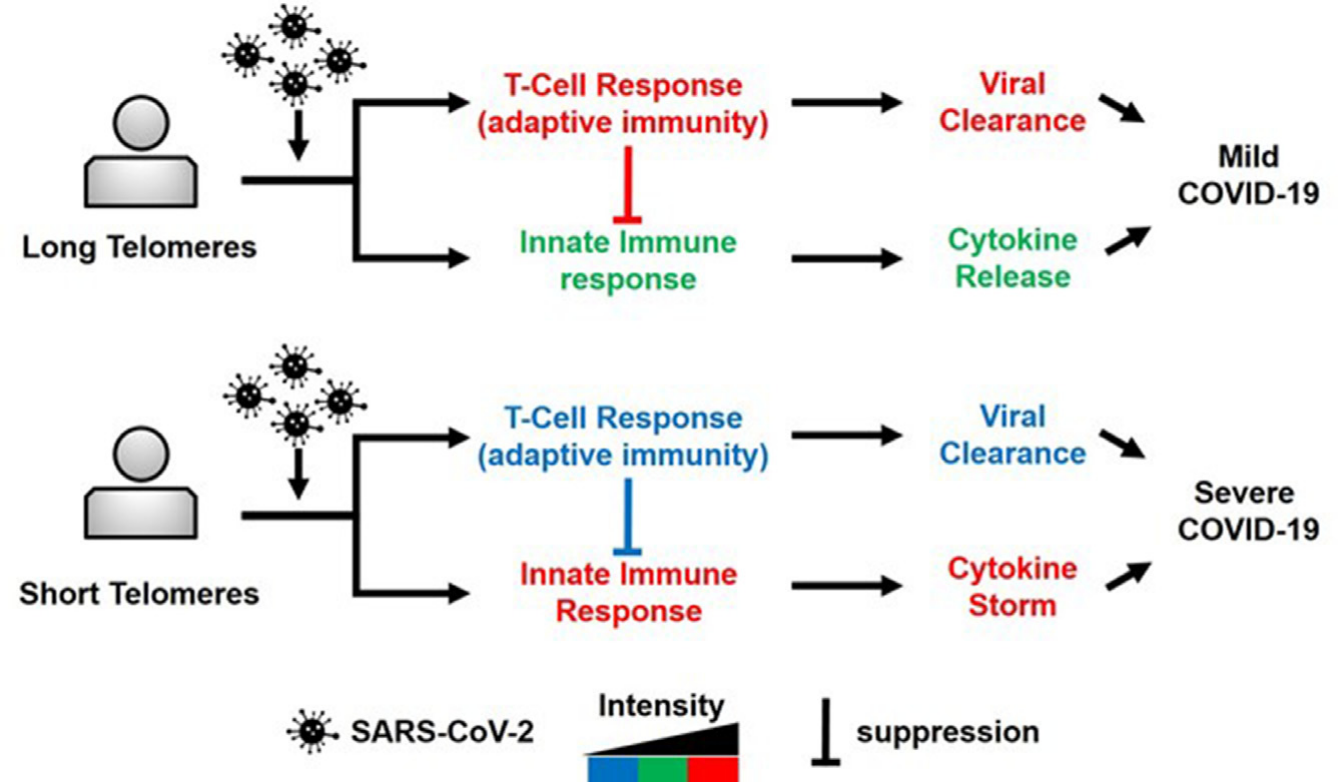
T-cell telomere length effect on the adaptive immune response and the innate immune response to SARS-COV-2 infection. For the individual with long T-cell telomeres, red color denotes strong T-cell response and robust suppression of the innate immune response. Green color denotes ‘calibrated’ (moderate) activity of the innate immune response. For the individual with short T-cell telomeres, blue color denotes weak T-cell response and inadequate suppression of the innate immune response. Red color denotes poorly ‘calibrated’ (strong) activity of the innate immune response, expressed in cytokine storm, lung injury and severe COVID-19.

TL of somatic cells reflects their inherent length that is determined at birth and their replicative history thereafter, explaining the shorter hematopoietic cell TL in older adults. However, the vast variation in LTL across the general population from birth onwards means that LTL of some adults in their thirties is as short as LTL of other adults in their eighties [9]. If TL plays a causal role in the immune response to SARS-CoV-2, these young adults with inherently short LTL might have a heightened risk to severe COVID-19. That said, COVID-19 mortality (due to severe disease) rises exponentially with age principally after the sixth decade of life, and in a recent theoretical work published in *medRxiv* (https://www.medrxiv.org/content/10.1101/2021.05.19.21257474v2), Anderson et al suggest that the T-cell response to SARS-CoV-2 through clonal expansion drops exponentially at approximately the same age. The TL-COVID-19 link might thus be defined by a TL “threshold”, such that TL minimally affects the course of COVID-19 until reaching this threshold. Once this threshold is crossed, largely after the sixth decade, short T-cell TL might have an amplifying, age-dependent effect on COVID-19 severity.

Finally, LTL was determined in the UK Biobank samples by qPCR, which generates data in relative T/S units and is the TL measurement- of- choice for population-based telomere research. However, the high measurement error of the method has raised escalating concerns. Using the UK Biobank telomere data, Wang's paper and its companions published elsewhere are templates for how to take advantage of the high throughput of the qPCR method to study large cohorts and thereby offset its high measurement error. The next step, however, i.e., deciphering the contribution of T-cell TL dynamics (TL and its shortening) to the development of T-cell lymphopenia and its aftermath, will require more precise and sequential measurements of T-cell TL that generate data in absolute units of TL in smaller cohorts of COVID-19 patients.

## Declaration of Competing Interest

The author declares no conflict of interest.
